# Intersecting Paths of Emerging and Reemerging Infectious Diseases

**DOI:** 10.3201/eid2705.204779

**Published:** 2021-05

**Authors:** Tais M. Wilson, Christopher D. Paddock, Sarah Reagan-Steiner, Julu Bhatnagar, Roosecelis B. Martines, Andrea L. Wiens, Michael Madsen, Kenneth K. Komatsu, Heather Venkat, Sherif R. Zaki

**Affiliations:** Universidade de Brasília, Brasília, Brazil (T.M. Wilson);; Centers for Disease Control and Prevention, Atlanta, Georgia, USA (T.M. Wilson, C.D. Paddock, S. Reagan-Steiner, J. Bhatnagar, R.B. Martines, H. Venkat, S.R. Zaki);; Pinal County Office of the Medical Examiner, Florence, Arizona, USA (A.L. Wiens);; Coconino County Health and Human Services Medical Examiner’s Office, Flagstaff, Arizona, USA (M. Madsen);; Arizona Department of Health Services, Phoenix, Arizona, USA (K.K. Komatsu, H. Venkat)

**Keywords:** coronavirus disease, COVID-19, severe acute respiratory syndrome coronavirus 2, SARS-CoV-2, hantavirus, Peromyscus maniculatus, Sin Nombre virus, hantavirus pulmonary syndrome, HPS, vector-borne infections, respiratory infections, pathology, histopathology, immunohistochemical analysis, co-infection, differential diagnoses

## Abstract

Severe acute respiratory syndrome coronavirus 2 (SARS-CoV-2) shares common clinicopathologic features with other severe pulmonary illnesses. Hantavirus pulmonary syndrome was diagnosed in 2 patients in Arizona, USA, suspected of dying from infection with SARS-CoV-2. Differential diagnoses and possible co-infections should be considered for cases of respiratory distress during the SARS-CoV-2 pandemic.

Severe acute respiratory syndrome coronavirus 2 (SARS-CoV-2), the virus that causes coronavirus disease (COVID-19), emerged in Wuhan, China, during December 2019 and spread rapidly to other parts of China and the world (*1*). However, the clinical and pathologic features of COVID-19 are also found for other respiratory disease, such as hantavirus pulmonary syndrome (HPS). In 1993, a hantavirus (Sin Nombre virus) and its rodent reservoir (*Peromyscus maniculatus* deer mouse) were identified as the causative agent and vertebrate reservoir responsible for an outbreak of severe pulmonary illness, named HPS, in the Four Corners region in the southwestern United States (*2**–**4*).

Soon after the emergence and recognition of COVID-19 in the United States in early 2020, the Infectious Diseases Pathology Branch, Division of High-Consequence Pathogens and Pathology, National Center for Emerging and Zoonotic Infectious Diseases, Centers for Disease Control and Prevention initiated diagnostic testing of fixed tissue specimens from deceased persons who had suspected or confirmed SARS-CoV-2 infection (*5**,**6*). During May 2020, Infectious Diseases Pathology Branch received tissues from an 11-year-old child (patient 1) from Arizona, who died after a brief illness culminating in severe respiratory distress. Histopathological findings included diffuse alveolar damage with rare hyaline membranes, intraalveolar edema, leukocytosis with a left shift (Figure, panel A), interstitial pneumonitis and immunoblasts in the red pulp and periarteriolar sheaths of the spleen (Figure, panel B). RNA extracted from formalin-fixed, paraffin-embedded trachea and lung tissues was positive for SARS-CoV-2 by conventional reverse transcription PCR (RT-PCR) and sequencing of positive amplicons. However, evaluation for SARS-CoV-2 by using an immunohistochemical (IHC) assay (*5*) showed negative results.

**Figure:**
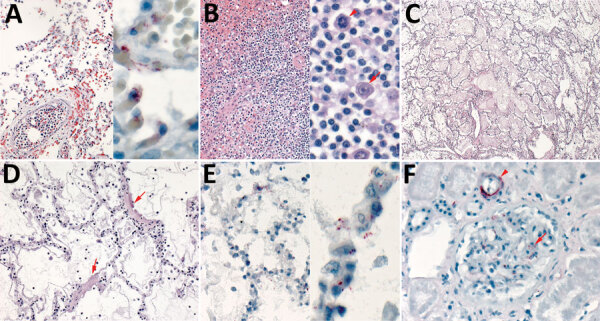
Histopathologic and immunohistochemical characteristics of fatal hantavirus pulmonary syndrome in 2 patients, Arizona, USA, 2020. A) Patient 1 lung tissue, showing intravascular leukocytosis with left shift (left, original magnification ×50) and hantavirus antigen immunostaining (red) in pulmonary microvasculature (right, original magnification ×158). B) Patient 1 spleen tissue, showing immunoblast proliferation in the red pulp and periarteriolar sheaths (left, original magnification ×50) and immunoblasts with high nuclear to cytoplasmic ratio, vesicular and prominent nucleoli (arrows) and mitosis (arrowhead) (right, original magnification ×158). C) Patient 2 lung tissue, showing severe intraalveolar edema (original magnification ×12.5). D) Patient 2 lung tissue, showing interstitial pneumonitis with hyaline membranes (arrows) (original magnification ×50). E) Patient 2 lung tissue, showing hantavirus antigen immunostaining (red) in pulmonary microvasculature (left, original magnification ×50; right, original magnification ×158). F) Patient 2 kidney tissue, showing hantavirus antigen immunostaining (red) in glomerular capillaries (arrowhead) and interstitial vessel (arrow) (original magnification ×100).

Subsequently, embalmed lung tissues were received from the child`s mother, a 25-year-old woman (patient 2) who died 2 days before the child after a brief illness characterized by progressive shortness of breath, cough, abdominal pain, fever, and hemoptysis. Histopathologic findings for the lungs of patient 2 resembled those identified for patient 1 (Figure, panels C, D), but there was no evidence of SARS-CoV-2 in the lung tissues by RT-PCR. Because clinicopathologic features were characteristic of HPS, we performed IHC assay for hantavirus. IHC showed typical punctate granular staining of hantaviral antigens in pulmonary and glomerular capillaries, characteristic of HPS (*4*) (Figure, panels E, F). IHC evaluation of lung and kidney tissues of patient 1 for hantavirus showed a similar pattern, confirming the infection in both patients (Figure, panel A, right side).

The clinicopathologic and IHC findings indicate that both patients died from HPS. Although SARS-CoV-2 RNA was detected by RT-PCR in patient 1, it was not the probable underlying cause of death. This scenario provides an essential reminder that previously recognized, nonendemic infectious diseases that clinically resemble COVID-19 continue to occur during the pandemic, in a manner similar to other clinicopathologic mimics described previously during other pandemic diseases (*7*).

Consideration of alternative diagnoses of diseases that precipitate acute respiratory distress syndrome and co-infections remains crucial for diagnosing and treating of critically ill patients, as well as accurately determining causes of death. For HPS, triage tools such as peripheral blood smear review and identifying 4 of 5 findings (thrombocytopenia, hemoconcentration, granulocytic left shift, absence of toxic changes, and >10% immunoblasts) can be used to diagnose the disease rapidly and presumptively in the clinical setting (*8**,**9*). Communication and partnerships of local, state, and federal public health officials and healthcare professionals, including clinicians, infectious disease specialists, pathologists, and medical examiners, are essential during these challenging times of the SARS-CoV-2 pandemic.
